# The Troubling Link Between Non-alcoholic Fatty Liver Disease (NAFLD) and Extrahepatic Cancers (EHC)

**DOI:** 10.7759/cureus.17320

**Published:** 2021-08-20

**Authors:** Ajit Venniyoor, Abdul Aziz Al Farsi, Bassim Al Bahrani

**Affiliations:** 1 Medical Oncology, National Oncology Center, The Royal Hospital, Muscat, OMN; 2 Oncology, National Oncology Center, The Royal Hospital, Muscat, OMN

**Keywords:** extrahepatic cancers, diabetes type 2, adipose tissue, nafld, fatty liver, cancer, insulin resistance, ectopic fat storage, obesity-related cancers

## Abstract

Non-alcoholic fatty liver disease (NAFLD) is a fast-spreading epidemic across the globe and has serious implications far beyond that of a “benign” liver condition. It is usually an outcome of ectopic fat storage due to chronic positive energy balance leading to obesity and is associated with multiple health problems. While association with cardiovascular disease and hepatocellular cancer is well recognized, it is becoming clear the NAFLD carries with it an increased risk of cancers of extrahepatic tissues. Studies have reported a higher risk for cancers of the colon, breast, prostate, lung, and pancreas. Fatty liver is associated with increased mortality; there is an urgent need to understand that fatty liver is not always benign, and not always associated with obesity. It is, however, a reversible condition and early recognition and intervention can alter its natural history and associated complications.

## Introduction and background

Non-alcoholic fatty liver disease (NAFLD) is one of the commonest indicators of ill-health in the modern era of obesogenic lifestyle, next to obesity itself [1,2]. It affects a quarter of the world’s population, with more than a billion individuals suffering from this condition worldwide [3,4]. NAFLD encompasses a wide spectrum of conditions ranging from “benign” fatty liver (hepatic steatosis, non-alcoholic fatty liver (NAFL), which is the accumulation of fat in more than 5% of hepatocytes), to non-alcoholic steatohepatitis (NASH, the inflammatory stage), to fibrosis, cirrhosis (with later decompensation), and hepatocellular carcinoma (HCC). The advanced stages are now the commonest cause of liver transplantation in the western world [5].

There are multiple causes for fatty liver, but the term NAFLD is conventionally restricted to that related to nutritional causes, obesity, and metabolic syndrome. Causes of fatty liver can be genetic (PNPLA3 mutation, lipodystrophies) [6], drugs [7], surgery (pancreatico-duodenectomy) [8], and hormonal (such as deficiency of thyroxine, estrogen, or testosterone) [9], but these are excluded while discussing NAFLD. There is a proposal to change the name from NAFLD to the more specific metabolic-associated fatty liver disease (MAFLD), which is scientifically accurate and removes the stigma associated with alcohol [10]. It must be noted that although NAFLD is thought to be a disease of the obese (reaching up to 50% in this population [11]), it is not necessary for a person to be overweight to have NAFLD (“lean NAFLD” [12]). However, it is clear that the vast majority of NAFLD is associated with metabolic and lifestyle disorders [13]).

## Review

Epidemiology

The prevalence of NAFLD is disturbingly high across the world, varying between 25% and 35% globally. The highest prevalence is reported from South America (31%) and Middle East (32%), followed by Asia (27%) and the USA (24%), while the prevalence is lowest in Africa (14%) [14]. The Middle East thus carries a heavy burden of NAFLD cases.

Recent figures suggest that more than 50% of the Omani population is overweight, and 30% have a body mass index (BMI) of more than 30% [15]. Neighboring countries such as the United Arab Emirates (UAE) and Saudi Arabia which are also struggling with a high burden of obesity have reported a NAFLD prevalence of 25% [16,17], posing a significant economic burden [18]. A recent analysis of Global Burden of Disease data noted the increasing prevalence of NAFLD across all regions of the world from 1990 to 2017 and pointed out Oman as the country with the maximum increase [19]. Similarly, NASH as a cause of cirrhosis in Oman increased from an age-standardized rate (ASR) of 3.54 in 1990 to an ASR of 9.59 in 2017, a 540% surge [20]. Among the 195 countries studied in this report, the highest growth rate of incident cases was found in the Gulf Cooperation Council countries with UAE leading with a whopping 1,119% increase, followed by Qatar (776%) and Oman (540.67%) [20].

Surprisingly, in a study of HCC diagnosed from Oman between 2008 and 2015, of 284 patients, only two of 227 patients with cirrhosis (0.9%) were reported to have a cryptogenic etiology [21] (potentially due to NASH), while alcoholic cirrhosis was much higher at 9.5%. This highlights a vexing problem; overlapping conditions such as viral hepatitis in the case of NASH will lead to underestimation of NASH as a cause of cirrhosis and under-recognition of NASH as a cause of HCC.

Mechanisms of NAFLD

Steatosis occurs in hepatocytes due to two main mechanisms [22] - increased accumulation which is due to the overflow of free fatty acids (FFAs) from adipocyte tissue (which contributes to 60% of the stored fat), increased de novo lipogenesis (25%) and FFA from the diet (15%-20%) and reduced output which is due to reduced FFA oxidation and reduced secretion of lipids. De novo lipogenesis is particularly affected by the consumption of fructose in “sugary” drinks [23].

The relation between obesity, hyperinsulinemia, insulin resistance, type 2 diabetes mellitus (T2DM), and NAFLD is a complex, “chicken and egg” situation. While the events that spark the inflammation that converts steatosis to steatohepatitis have been comprehensively reviewed [24], what causes the initial fat accumulation (simple steatosis) and insulin resistance is a matter of controversy. Few authors believe that insulin resistance causes NAFLD [25,26] while many suggest that NAFLD leads to insulin resistance and hyperinsulinemia [27,28]. The association is clear; a recent meta-analysis showed that NAFLD is associated with a 2.2 increased risk of T2DM [29]; the question is which comes first. This discussion is intimately tied to the controversy over the origins of obesity. The older “calorie in, calorie out” (CICO) or energy balance model is being challenged by the carbohydrate-insulin model (CIM) of obesity [30]. Current evidence suggests that constant positive energy balance in the modern obesogenic environment leads initially to hyperinsulinemia [31] in an effort to maintain normoglycemia. Indeed, it is known that insulin levels rise almost a decade before diabetes is detected [32-35] and are sometimes referred to as Stage 1 of diabetes associated with compensation [36].

Insulin is an anabolic, fat-storage hormone, and hyperinsulinemia results in varying degrees of obesity depending on the fat storage capacity of the individual (the “personal fat threshold” [37]). The latter is determined genetically or in utero. Fat is initially stored in the “safe” white adipose tissue (WAT), which is predominantly subcutaneous. Chronic positive energy intake leads to the excess fat (in form of FFAs) spilling over; the extra fat is stored in “unsafe” sites such as visceral adipose tissue (VAT) and liver (NAFLD) (ectopic fat storage). The outcome is insulin resistance (by lipid metabolites like diacylglycerol and ceramides), and T2DM [31]. NAFLD is further aggravated by fructose-containing “sugary” foods [23,38]. 

It is clear now that hyperinsulinemia is one of the earliest manifestations of modern-day chronic calorie excess/adiposity and appears much before the onset of insulin resistance and T2DM [39]. The modern high-carbohydrate diet with a high glycemic index and glycemic load is directly responsible for the raised insulin levels. Several studies have shown that low carbohydrate intake (which results in a ketogenic or “insulin sparing” diet) can reverse these changes and indeed, T2DM itself [40]. It is probable that fatty foods have been wrongly blamed for the obesity epidemic, and the sugar [41] and fast-food industry marketing ultra-processed food [42] have actively contributed to this myth [43].

The hyperinsulinemic syndrome [44] has other manifestations, including cardiovascular, respiratory, and renal disorders, cancer, and reproductive diseases such as polycystic ovarian syndrome (PCOS); these can occur independently of obesity or T2DM. It is not clear whether these diseases are due to high levels of insulin per se, or other proximate causes.

NAFLD and extrahepatic cancers (EHC)

It was conventionally thought that the earliest manifestation of NAFLD, the fatty liver (NAFL), is a benign condition and becomes a medical problem only when it progresses to NASH, and later to fibrosis, cirrhosis with decompensation, and/or HCC. However, recent evidence suggests that the finding of hepatic steatosis is not benign and has significant health consequences [45]. NAFLD is an early warning indicator of extrahepatic disorders involving the cardiovascular [46], respiratory [47], renal [48], and reproductive system [49]. NAFLD is also associated with an increased risk of premalignant conditions such as colorectal polyps [50] but apparently not with ductal carcinoma in situ (DCIS). Several recent publications have now highlighted the risk of EHCs, something that has been underestimated so far [51].

In a prospective study from Rochester County, Minnesota, Allen et al. [52] followed 4,722 patients of NAFLD for a median of eight years and reported a 90% higher risk of malignancy (2,224 incident cancers; RR 1.9), especially of liver, endometrium, stomach, pancreas, and colon. Surprisingly, obesity alone did not increase the relative risk (consistent with the idea that people with adequate safe storage space for fat may become obese but will remain healthy, the metabolically healthy obese or MHO). The authors suggest that as the association between obesity and cancer risk is small; NAFLD may be a mediator of the obesity-cancer association.

In a long-term population-based cohort study from Sweden of 8,892 patients of biopsy-proven NAFLD [53], EHC was reported as a major cause of mortality. There is a higher risk of HCC across the spectrum of simple steatosis to cirrhosis as expected, and also a modest rise in EHCs (that of the pancreas, kidney/bladder, and melanoma)

A meta-analysis by Mantovani et al. [54] of 10 cohort studies with 8,485 incident cases of EHCs occurring over a median follow-up of 5.8 years reported that NAFLD was associated with a 1.5-fold to twofold risk of GI cancers (esophagus, stomach, pancreas, and colorectal cancers), and 1.2-fold to 1.5-fold risk of lung, breast, gynecological and urinary system cancers. (Although lung cancer is not what one would expect in relation to obesity, a Chinese study [55] did report that both obesity and NAFLD are independent risk factors for adenocarcinoma of the lung, as did a Danish study [56].)

Another meta-analysis of 26 studies [57] has confirmed the association of NAFLD with EHCs, especially of colon, breast, esophagus, stomach, pancreas, prostate, and also cholangiocarcinomas.

 A decade-long study from China [58] also showed that a significant cause of mortality in NAFLD is due to EHCs, second only to cardiovascular causes. In this study of more than 10,000 deaths during hospitalization, cardio- and cerebral vascular disease (CVD) ranked first in causes of death (35.6%), followed by extrahepatic malignancies (22.6%), infection (11.0%), kidney disease (7.5%), liver-related diseases (5.2%), respiratory diseases (3.9%), digestive diseases (3.5%), endocrine diseases (3.5%), and other diseases (7.2%). 

What is interesting is that obesity is not an essential requirement for EHCs associated with NAFLD. Obesity-related cancers (ORCs) are a recognized entity [59], and the International Agency for Research on Cancer report in the New England Journal of Medicine listed 13 cancers with a higher incidence in obese individuals, especially that of the GIT and the reproductive system [60]. It is well known that BMI, which IARC used as its criteria, is an inadequate measure of unhealthy obesity, as it neither indicates the percentage of body fat mass nor the location of the fat [61], which determines the metabolic impact. Individuals with normal BMI but with “metabolic obesity” (sometimes called TOFI; thin outside, fat inside, an allusion to visceral obesity) have a higher cancer risk [62]. People at the opposite end of the spectrum, i.e., obese but apparently healthy (the MHO) are now noted to have excess mortality mainly due to cardiovascular causes; an excess risk of cancers has not been reported [63].

In Allen’s study [52], NAFLD was associated with a higher risk of incident cancers (overall malignancy IRR=2.0), while obesity alone was not (IRR=1.0). Ectopic fat deposition, which cannot be measured by BMI, seems to be the common underlying factor in the etiopathogenesis of metabolic disorders and metabolic cancers.

Association is, of course, not causation and the larger question is, does fatty liver directly cause cancer? Causality is difficult to establish, and there could be another proximate cause for both fatty liver and cancer. Reversal of NAFLD leads to a drop in the incidence of cancers, the most effective way being bariatric surgery [64]. It is known that weight loss of 5% reverses NAFLD. However, the Women Health Initiative (WHI) study indicates that while an intentional 5% weight loss reduced overall cancer risk (HR 0.88), and specifically that of endometrial cancer (HR 0.72), it did not reduce the risk of other ORCs such as of colon, breast, pancreas, kidney, thyroid, or liver [65]. The ultimate proof will probably come from long-term follow-up of patients specifically treated for NAFLD without other metabolic perturbations; this should indicate whether the ultimate cause lies in the liver or in the adipose tissue.

Mechanisms of carcinogenesis

The molecular links between obesity and cancer have been speculated on [66]; recent reviews have addressed the molecular mechanisms tying NAFLD to HCC [67] but the remote effects of NAFLD leading to EHC are less clear. The hormones, insulin and insulin-like growth factor-1 (IGF-1) have been implicated with some evidence [68].

The liver itself secretes a vast repertoire of hepatocytes [69,70], some of them with proliferative potentials such as FGF21 and fetuin-A [71]. To add to the complexity, several cytokines such as irisin [72] (mainly from skeletal muscles, another site of ectopic fat deposition), and leptin and adiponectin [73] (from adipose tissue) can alter tumor growth.

The mechanism(s) is much more than of academic interest as blocking the proliferative/mutagenic signals should (theoretically) uncouple NAFLD from its remote effects such as EHCs. This should be an area of research priority as it is unlikely that efforts to curb the incidence of NAFLD will bear fruit in near future. 

Implications

Effect on Natural History

It is an intriguing question whether the presence of NAFLD alters the natural history of cancer. Some studies suggest that fatty liver increases the risk of liver metastasis (in lung cancer) [74], but others indicate no such effect (rectal cancer) [75] or even a reduced risk (in breast and colon cancer) [76,77].

Effect on Drug Response

Another evolving issue is the altered dynamics of immune checkpoint inhibitors (ICIs), in patients with NAFLD. ICIs themselves have been reported to cause NAFLD [78] and also cause drug-induced liver injury (DILI) in patients of NAFLD [79]; obese patients treated with ICIs also have a higher risk of thyrotoxicosis [80]. The better survival of some cancer patients (renal cell, melanoma, lung) if associated with obesity has been termed an “obesity paradox” [81]; this effect is consistent with a better response to ICIs in obese renal cell cancers patients [82]. However, a recent meta-analysis concluded that the current evidence does not support a clear positive association of BMI with survival outcomes [83]. Response to ICIs may be cancer-specific and even metastatic-site specific; one study concluded that HCC arising in a background of NASH is less responsive to ICIs [84], but liver metastasis of lung cancer responded better in NASH-affected livers [85]. Interestingly, liver metastasis from colorectal cancer has a higher risk of recurrence if resected from NASH-affected livers [86]. Further studies are needed but, in the meantime, Oncologists should be cautious while using this class of drugs in patients with NAFLD in view of a higher reported risk of DILI.

High-Risk Warning

At the general practitioner level, there should be increasing awareness that hepatic steatosis is not a benign condition; it should not be dismissed as an incidental finding in the ultrasound scan of the abdomen, as is done for renal or liver cysts. Firstly, about 20% of NAFL progress to NASH, and 40% of the latter go on to develop fibrosis; the risk of progression to NASH and fibrosis can be rapid, with NAFL progressing to fibrosis in an average of 14 years while NASH can in half that duration [2]; this can be dramatic in some. Secondly, identification of NAFLD on ultrasound (or by the asymptomatic rise in the liver enzyme ALT) demands to work up for associated conditions such as hypertension, T2DM, dyslipidemia, and other components of metabolic syndrome [2]. The higher risks of not just HCC but also of EHCs should be informed to the patient. The workup of such patients has been standardized and liver biopsy may be indicated in selected patients [1]. There should be an all-out effort to reverse it, starting with lifestyle changes, the simplest being to avoid sugary drinks. “Lean NAFLD” is not common and obesity remains overwhelmingly the largest cause of NAFLD. Despite social trends to avoid fat-shaming and accept obesity as the “new normal” [87], as doctors we must continue to campaign vigorously against obesity, as we did against smoking. This should include supporting legislation to make sugary and ultra-processed foods expensive and less accessible while subsidizing healthier options.

Recommendations for Screening?

It has been suggested that patients with NAFLD should be candidates for targeted screening for EHCs [52]. Three of the commonly associated cancers, that of colon, prostate, the breast are already in screening guidelines of many countries, and there should be an effort to include more in patients of NAFLD, such as endometrium. Prospective studies are required in this setting - especially to study the cost-effectiveness of screening and whether this leads to reduction of mortality - the only meaningful endpoint of screening studies.

Management

Reversal by Weight Loss

Reversal of NAFLD can either be by weight loss or by specifically targeting NAFLD. Weight loss through lifestyle modification (diet and exercise) is the most cost-effective intervention to reverse NAFLD [88,89] but decades of effort and billions of dollars later, it is clear that weight loss by dieting is easier said than done. Moderate weight reduction is highly effective at decreasing hepatic fat content [90], but 70% to 95% of those who lose significant weight subsequently regain it [91]. The Endocrine Society’s statement [92] on the pathogenesis of obesity noted that the recovery of lost weight is the largest single obstacle to effective long-term weight loss and identifying biological mechanisms that defend excess fat is a priority. Bariatric surgery remains the single most effective intervention to reverse obesity and its metabolic consequences including NAFLD and is associated with reduced risk of cancers including cancers of colon, pancreas, endometrium, thyroid, and of course, liver [93]; however, this procedure cannot be recommended for all as it comes with known morbidity and mortality, is cleared only for the morbidly obese and fails in about 15% of patients. The effects of bariatric surgery can be mimicked by the very low-calorie diet (VLCD) as shown by the DIRECT trial [94]. Adapting to a Mediterranean diet is a viable option to prevent and treat NAFLD [95]. GLP-1 receptor agonists such as liraglutide [96] and semaglutide [97] can produce up to 10%-15% weight loss and are increasingly used to manage NAFLD [98].

Targeted Reversal

Specific treatment of NAFLD is in its nascent stages [99]. It is clear that weight loss is not necessary to reverse NAFLD; NAFLD can be addressed with a low carbohydrate, high protein, high fiber diet [100,101], or fructose-limited diet [102]. Despite active research [103,104], there are very few pharmacological interventions in current guidelines [105]. Pioglitazone, vitamin E, and ursodeoxycholic acid (UDCA) are possible agents but their safety is a matter of concern [106]; more are in the pipeline [107].

An epidemiological study from China [108] gives indirect evidence that personal capacity of fat storage is related to the risk of NAFLD. Individual fat storage capacity is determined by the time of reaching adulthood and this study showed that exposure to famine conditions during the fetal or childhood stage (with minimal or no development of fat storage capacity) leads to a higher risk of NAFLD in adulthood. The implication is that promoting healthy energy storage in the face of caloric excess may lead to an uncoupling of obesity from NAFLD and the metabolic syndrome [109]. Expansion of subcutaneous “safe” WAT is a method of diverting fat away from the liver; though causing weight gain, this technique normalizes metabolic parameters and reverses NAFLD associated with obesity. This has been demonstrated experimentally in adiponectin overexpressing transgenic mice [110]. Pioglitazone [111,112] and other PPARγ agonists act similarly and are effective in NAFLD; however, as can be anticipated, they cause weight gain.

Several agents that directly reverse NAFLD such as controlled-release mitochondrial protonophore (CRMP) are being studied [113]. Two rather unusual approaches are fecal microbiota transplantation [114] and whole-body vibration [115].

Finally, research is essential to discover the mechanisms linking NAFLD to EHCs so as to enable intervention. It is unlikely that we will achieve any form of control over the burgeoning obesity and NAFLD rates in near future [116]. The prevalence of NAFLD will continue to rise [117]. NAFLD is fueling an upsurge in cardiovascular diseases (CVD) [118] including hypertension [119]. Although a similar rise in T2DM has the potential to increase CVD, a recent study from the UK showed that death rates due to vascular diseases are actually declining in patients of T2DM (leaving cancer as now the leading cause of mortality) [120]. This was achieved not just by better glycemic control but by controlling other risk factors for vascular diseases, such as hypertension and dyslipidemia. Similarly, intense research is needed to identify additional risk factor(s) linking NAFLD to EHC, which can then be addressed.

## Conclusions

Chronic positive energy balance overwhelms safe fat storage depots in subcutaneous WAT, leading to ectopic fat storage in unsafe sites such as VAT and liver. This causes insulin resistance, metabolic syndromes, and, as is increasingly clear, EHC. Clinicians and their patients should be made aware that fatty liver is just the tip of an iceberg; NAFLD is an indicator of an underlying plethora of metabolic disturbances. Aggressive measures must be instituted early to reverse it. Obesity and NAFLD will continue to rise and will overwhelm the health care system in a not-too-distant future. Current advice on the control of obesity such as lifestyle changes is a clear failure. It would be prudent to investigate the causative link(s) between NAFLD and EHCs with the ultimate aim of discovering methods to uncouple them.
